# Children-related factors associated with life satisfaction of urban older adults living alone in China

**DOI:** 10.3389/fpubh.2024.1353052

**Published:** 2024-02-14

**Authors:** Rong Zhou, Jinghang Cui, Xingxing Yin

**Affiliations:** ^1^College of Philosophy, Law and Political Science, Shanghai Normal University, Shanghai, China; ^2^School of Physical Education, Jiangsu University of Science and Technology, Zhenjiang, China; ^3^Center for Aging and Health Science, Jiangsu University of Science and Technology, Zhenjiang, China; ^4^School of Social Development, East China Normal University, Shanghai, China

**Keywords:** number of children, gender structure, gender of children by birth order, life satisfaction, urban older adults living alone

## Abstract

**Introduction:**

The aim of this study is to investigate the association between the number of children, their gender structure, and the gender of children by birth order with the life satisfaction of urban older adults living alone (UOALA) in five Chinese cities. Traditional reproductive views suggest that having more children, especially sons, is associated with higher life satisfaction for older adults.

**Methods:**

This study utilized a cross-sectional design and included a sample of 2,801 UOALA from five Chinese cities. The life satisfaction of participants was measured using standardized questionnaires. To analyze the data, both OLS and OLogit methods were employed.

**Results:**

Empirical research shows that the number of children has a positive association with life satisfaction of UOALA, which is greater in male older adults than in female ones. The increase in the number of daughters is significantly associated with higher life satisfaction. In terms of gender structure, UOALA with multiple children, including both sons and daughters, tend to have a higher level of life satisfaction, which partly validates the cohort reproductive preference. In terms of gender of children by birth order, UOALA whose first child is a daughter have higher life satisfaction, which is more pronounced among male UOALA, while the association of gender of children by birth order on female UOALA is relatively weak.

**Discussion:**

This study contributes to the understanding of the factors associated with the life satisfaction of UOALA in China. The findings suggest that having more children, particularly daughters, and a balanced gender structure of children is associated with higher levels of life satisfaction. The study suggests the need for targeted social support for UOALA with varying family structures.

## 1 Introduction

Given the accelerating process of population aging and the increasing complexity of the aging issue, living conditions, quality of life, and subjective well-being of older adults have become increasingly of concern. Among them, life satisfaction is an important component of “subjective well-being” as an indicator (1). Influenced by the traditional view of “eldercare by family members” and “filial duty”, children are still the main responsible party for eldercare in China. The various intergenerational support provided by children has a profound association with the life satisfaction of the older in their later years. However, there is a lack of consensus regarding the relationship between children and the life satisfaction of older individuals. This is especially true for older individuals who live alone, as research specifically focusing on this group is particularly insufficient. This paper pays attention to this topic mainly for two reasons. Firstly, UOALA are a vulnerable group among the older adults population, as they tend to lack sufficient companionship from family members, which can lead to a lower life satisfaction. Secondly, with the trend of smaller family sizes and changes in living arrangements consequent upon urbanization, the number of older adults living alone will continue to increase. Hence, it is crucial to study the life satisfaction of these individuals. Given the one-child policy from the 1970s till 2015, most older people would have only one child within the next decade. Hence, using this finding to promote the two-child and three-child policies will be more meaningful as China faces a low fertility crisis.

Existing literature has primarily focused on the study of the gender of children, in topics such as eldercare resources (2), reproductive preferences (3), and family income (4). The measurement indicators often include the number of children, whether they have sons or daughters, the ratio of sons to daughters, and the gender of the first child (5–7). Vu et al. (8) find that there is an association between the number of children and a range of physical and psychological health indicators in parents. Parents with well-educated children exhibit higher levels of life satisfaction. Artamonova et al. (9) discover a relationship between the gender, quantity, and proximity of adult children and the relocation or institutionalization of their older adult parents. Zheng et al. (10) confirm that the financial status of adult children has a notable direct link with the happiness of the older adults population, even exceeding the associations with children’s education level and intergenerational support. While many studies have considered the differences in family size and economic level, they have not comprehensively considered the gender of children by birth order, leaving room for further exploration. Gu (11) has proposed that the study of fertility issues should consider the dimensions of quantity, timing, and gender. Therefore, this study attempts to analyze the association of the number of children, their gender structure and gender of children by birth order with the life satisfaction of UOALA. In contrast to rural areas where the reliance on labor input and the dominance of males are prominent, the urbanization process may have different influences on the older population due to the traditional cultural context of “raising children for old age” and the view of “more children, more blessings.” The research findings of this study provide insights into the association of fertility on the later life of older parents and serve as a reference for exploring reproductive life planning among the reproductive-age population in urban areas, amidst the background of continuous adjustments in fertility policies.

## 2 Research design

### 2.1 Research hypotheses

The theory of population and the theory of fertility utility argue that having children contributes to happiness (12), and the traditional view of “raising children for old age and accumulating grain for famine prevention” has a profound influence in China as well. This is because the traditional system of family old-age support is based on the intergenerational exchange of resources within the family, and the number of children is a condition for discussing the ability of family old-age support. In other words, the functionality of old-age support relies mainly on the “feedback” from children (13). An increase in the number of children can prove a more solid economic support and material and human resources for life care (14), making people more inclined to have more children to obtain family security in old age. This study focuses on the population born before the 1940s, who have a deep attachment to traditional values regarding the number of children and may be heavily influenced by the traditional view of “more children, more blessings.” Chen et al. (15) and Cao (16) find that the desire for fertility and the association with traditional beliefs are more prevalent in males compared to females, as males are not biologically involved in pregnancy and childbirth. Therefore, Hypothesis 1 is proposed as follows.

*H1:* Other things being equal, the greater the number of children, the greater the life satisfaction of UOALA. This may be more pronounced in male UOALA.

Research by Hou et al. (17) shows that having “both sons and daughters” has always been the top fertility preference for Chinese people. However, for parents in their old age, the traditional living arrangement of “marrying into the husband’s family” in China means that women are primarily responsible for supporting the elders in their husband’s family, thus the level of support provided by daughters to their parents is relatively reduced. However, with the progress of modernization, the advancement of women’s family and social status has resulted in the strengthening of their identity as “daughters” and the emergence of the characteristics of dual-caregiving for the older. On the other hand, the view of favoring sons over daughters stems from the ancient times when men had higher economic value in agriculture, thus the stability and prosperity of the family are closely linked to male members. With the advancement of technology and the adjustment of industrial structure, an increasing number of economic activities no longer rely on physical labor, resulting in a relative decline in men’s economic status. On the other hand, the increase in daughters providing assistance and emotional support to their older parents is significantly associated with higher life satisfaction. Based on these observations, Hypothesis 2 is proposed.

*H2:* Other things being equal, life satisfaction is higher among UOALA with both sons and daughters.

In exploring numerous factors that influence the life satisfaction of UOALA, this study also focuses on the role of different genders and birth orders of children. Asia, particularly East Asia, exhibits a clear preference for male offspring (18). According to distinctive criteria for evaluating lifestyles, the traditional Chinese culture harbors the notion of “favoring sons over daughters” and “elevating men while belittling women” when it comes to fertility beliefs (19). Zheng (20) discovers that after achieving the desired number of children, couples of childbearing ages are more likely to continue reproducing if they do not obtain the desired gender structure. Due to the traditional caregiving culture and gender perspectives that favor sons, influenced by the idea of “continuing the family line,” Chinese parents may experience a stronger sense of happiness by first giving birth to a son. Based on this, Hypothesis 3 is proposed as follows.

*H3:* When the total number of children and the gender composition are the same, UOALA whose first child is male tend to experience higher levels of life satisfaction.

### 2.2 Data sources

This study utilizes data from a project granted by the National Social Science Fund of China: “Research on the Home-Based Care System for the Urban Older Adults in China in the next ten years” conducted by the project team in Chengdu, Hohhot, Dalian, Guangzhou, and Shanghai from 2015 to 2016. The survey focuses on the living conditions and needs of older adults aged 70 and above who have lived in the respective areas for at least six months. The specific sampling method involves the following steps: Firstly, in each city, representative administrative districts such as old town areas, general urban areas, and new urban areas are selected from the city center or main urban area. If a surveyed city only has four or fewer administrative districts, all of them are included in the sampling. Secondly, within each selected administrative district, two randomly chosen streets are selected. Thirdly, within each street chosen, two randomly selected residential committees are chosen. Lastly, within each residential committee, 50 UOALA are sampled according to the customary house numbering sequence, from the front to the back. If a residential committee has less than 50 UOALA aged 70 and above, an equivalent number can be added by sampling from other residential committees. The survey employs a stratified sampling method and a multi-stage PPS method, covering 21 districts in the five cities and a total of 54 streets (towns). The survey consists of a long questionnaire and a short questionnaire, with the long questionnaire including all the questions from the short questionnaire. This paper uses the data from the long questionnaire, which was further checked and supplemented, resulting in a total of 2801 samples. In the given sample, 3.05% of the respondents do not have any children, while 12.18% have one child, 30.15% have two children, 32.22% have three children, and 22.41% have four or more children. Among these respondents, a notable 64.32% stated that they have both sons and daughters.

### 2.3 Research methodology

Based on the research hypotheses and cross-sectional survey data, this paper examines the association of the number, structure and order of children with the life satisfaction of UOALA and constructs the following model:

$$\log\frac{P\left(Y\leq j|x\right)}{1-P\left(Y\leq j|x\right)}=a_{j}+\beta^{\prime }x,j=1,2,3,4,5$$

To test whether there are marginal effects of the number of children and their gender composition on the life satisfaction of UOALA, it is necessary to further calculate the expression of the marginal effects of the explanatory variables. Typically, this involves calculating how the probability of the dependent variable taking different values is in relation to the referenced category when all explanatory variables are at their mean values (21). The equation is as follows:

$$\frac{\partial P\left(y=i|x\right)}{\partial x}|x=\bar{x}\left(i=1,2,3,4,5\right)$$

The dependent variable of this study is life satisfaction (sati). The questionnaire surveyed respondents’ self-assessment of their current life situation, asking “Are you satisfied with your current life conditions?” with values ranging from “very dissatisfied” to “very satisfied” assigned as 1 to 5. The higher the numerical value, the greater the life satisfaction with their current life situation. On the whole, respondents are generally content with their current lives, with over 70% of them stating they are either “fairly satisfied” or “very satisfied” with their current life conditions.

The explanatory variables for children include the number of children, their gender structure and gender of children by birth order. In terms of the number of children, it represents the total number of currently living children for the respondents. The gender structure of children refers to the quantity structure of sons and daughters among the respondents’ current children, with “M” representing sons and “F” representing daughters. For example, if there are 2 sons and 1 daughter, it would be represented as “2M1F”. If the first child is a son, the second child is a daughter, and the third child is a son, it would be represented as “MFM”. If two or more children are the same age, sons are labeled first. This article only discusses up to four children based on the distribution of the samples. If respondents have five or more children, only the first four children will be considered.

Previous research suggests that gender, age, residency, educational level, marital status, economic income, and health level have a significant association with the subjective sense of happiness in old age, leisure activities, and eldercare capital (2). Therefore, this paper selects gender, age, residency, migration status, educational level, marital status, average monthly income, self-rated health, and self-rated loneliness as control variables, as measured and assigned in Table 1. The number of children and gender of children by birth order are shown in Table 2.

**Table 1:** Settings, definitions and measurements of variables.

Variables	Options (as in computer software settings)	Sample size	Average value	(Statistics) standard deviation
Sati	Very dissatisfied = 1, Dissatisfied= 2, Neutral= 3, Satisfied = 4, Very Satisfied = 5	2746	3.87	0.83
Count	Number of children, self-administered by the respondent	2715	2.70	1.28
Gender	Gender structure of children	2607	6.41	3.78
Chorder	Child order structure	2607	9.85	8.09
Male	Male = 1, Female = 0	2801	0.32	0.47
Age	Age, self-administered by the respondent	2801	78.47	5.36
Hukou (residency)	Non-agricultural households = 1, agricultural households = 0	2779	0.95	0.22
Imm	Migrated = 1, not migrated = 0	2771	0.56	0.50
Education	Illiterate or very little literacy = 1, Elementary school = 2, Middle school = 3, High school = 4, Associate degree= 5, Bachelor’s degree and above = 6	2776	2.63	1.32
Mar	With spouse = 1, other = 2	2763	0.16	0.37
Income	Below ¥500 = 1, ¥500 to ¥999 = 2, ¥1,000 to ¥1,499 = 3, ¥1,500 to ¥1,999 = 4, ¥2,000 to ¥2,499 = 5, ¥2,500 to ¥2,999 = 6, ¥3,000 to ¥3,999 = 7, ¥4,000 to ¥4,999 = 8, ¥5,000 and above = 9	2789	5.03	1.86
Health	Very good = 1, Good = 2, Neutral= 3, Bad = 4, Very bad = 5	2706	2.97	1.06
Alone	Never = 1, sometimes = 2, often = 3	2661	1.81	0.72

**Table 2:** The percentage of number of children and gender of children by birth order.

Number of children	Gender of children by birth order	Full sample	Male	Female	Number of children	Gender of children by birth order	Full sample	Male	Female
One	M	7.58	9.3	6.75	Four	MMMF	1.54	1.19	1.70
	F	6.29	6.0	6.39		MMFM	0.88	0.79	0.91
Two	MM	8.42	7.94	8.64		MFMM	0.54	0.53	0.55
	FF	6.63	8.07	5.96		FMMM	0.96	0.53	1.16
	MF	12.25	15.08	10.95		MMFF	1.67	1.72	1.64
	FM	6.79	7.28	6.57		MFMF	0.50	0.53	0.49
Three	MMM	3.79	2.51	4.38		MFFM	0.71	0.53	0.79
	MMF	5.79	5.03	6.14		FMMF	0.88	1.19	0.73
	MFM	3.833	3.17	4.14		FMFM	0.79	0.66	0.85
	FMM	4.33	4.37	4.32		FFMM	1.25	0.93	1.40
	MFF	5.46	5.16	5.60		MFFF	1.33	1.19	1.40
	FMF	4.29	4.50	4.20		FMFF	0.58	0.40	0.67
	FFM	5.67	5.16	5.90		FFMF	0.92	1.06	0.85
	FFF	2.92	3.31	2.74		FFFM	1.33	0.79	1.58
Four	MMMM	0.96	0.53	1.16		FFFF	1.13	0.40	1.46

## 3 Empirical findings

### 3.1 The association of the number of children with life satisfaction

According to the research Hypothesis, Table 3 presents the estimated results of the association of the number of children with the life satisfaction of respondents, without considering the gender and order structure of the children. According to the regression results, an increase in the number of children has a positive association with the respondents’ life satisfaction. The increase in the number of children confirms the research Hypothesis. Columns (1) to (3) display the estimates obtained from the model for the full sample, males, and females respectively. The results for the full sample indicate a significant positive association of the number of children with respondents’ life satisfaction at the 1% significance level, with a coefficient of 0.117. To further examine the association of the number of children with the life satisfaction of respondents of different genders, separate regressions are conducted for each gender. The regression results remain significant. The number of children has a significant positive association with male respondents’ life satisfaction at the 1% significance level, and a significant positive association with female respondents’ life satisfaction at the 5% significance level, with coefficients of 0.233 and 0.081 respectively. In traditional Chinese culture, children play a significant role in the older care process. The view of “raising children for old age” and “more children, more blessings” is deeply rooted. Children’s economic support, emotional support, and spiritual support are significantly associated with life satisfaction. The more children one has, the more support resources they are likely to receive, which helps to enhance respondents’ life satisfaction.

**Table 3:** Regression analysis of the number of children of respondents with life satisfaction.

	Ologit		
	Full sample	Male	Female
	(1)	(2)	(3)
Sum	0.117***	0.223***	0.081**
	(0.035)	(0.069)	(0.040)
Male	-0.254***	-	-
	(0.088)	-	-
Age	0.016*	0.013	0.018
	(0.009)	(0.017)	(0.011)
Hukou	0.379*	0.640	0.307
	(0.197)	(0.469)	(0.213)
Imm	0.320***	0.366**	0.303***
	(0.083)	(0.163)	(0.098)
Education	0.033	0.048	0.027
	(0.036)	(0.068)	(0.044)
Mar	-0.081	-0.068	-0.088
	(0.109)	(0.192)	(0.135)
Income	0.120***	0.091*	0.126***
	(0.029)	(0.052)	(0.036)
Health	-0.490***	-0.631***	-0.437***
	(0.044)	(0.089)	(0.051)
Alone	-0.655***	-0.765***	-0.622***
	(0.065)	(0.133)	(0.075)
City	control	control	control
Cut1	-4.436***	-4.378***	-4.372***
	(0.775)	(1.438)	(0.943)
Cut2	-2.875***	-3.265**	-2.575***
	(0.754)	(1.399)	(0.914)
Cut3	-0.666	-0.834	-0.436
	(0.749)	(1.390)	(0.906)
Cut4	1.834**	1.910	1.979**
	(0.748)	(1.388)	(0.905)
N	2366	695	1671
R2			
F			
R2_p	0.077	0.113	0.066
Chi2	394.13	158.406	243.481

A two-tailed test is employed. The robust standard errors within parentheses, *, **, and *** represent significance levels of 10%, 5%, and 1% respectively. The same applies to the table below.

Furthermore, variables such as gender, age, residence, migration, income, health condition, and feelings of loneliness are significantly associated with life satisfaction. The results indicate that female respondents have higher life satisfaction levels compared to male respondents, passing the significance test at the 1% level. Age has a positive and significant association with life satisfaction, with respondents feeling more satisfied with life as they grow older. However, this association is not significant for male respondents. Non-rural residency is associated with higher levels of life satisfaction compared to rural residency, although this difference is not statistically significant when considering gender-specific samples. Migration is significantly associated with life satisfaction at the 1% level, suggesting that respondents who have experienced migration have higher levels of life satisfaction compared to those who haven’t. The purpose of migration may be to seek a better life, which in turn leads to higher levels of life satisfaction. Income also has a positive association with life satisfaction, with respondents who have higher incomes reporting higher levels of life satisfaction. Income serves as the fundamental guarantee for older adults’ lives, and those with higher economic levels have a greater ability to enjoy a better life, resulting in higher life satisfaction. Life satisfaction is closely related to health conditions, with self-rated health positively associated with life satisfaction at the 1% level. The better the physical condition, the higher the level of life satisfaction. Additionally, the experience of loneliness has the deepest association with life satisfaction. Respondents who experience intense loneliness tend to have lower life satisfaction levels, and this variable has the largest coefficient among all the variables.

### 3.2 Marginal effects of number of children with life satisfaction

To gain a deeper understanding of how the number of children is associated with the life satisfaction level of the respondents’ lives, the marginal effects of the number of children are calculated. An increase in the number of children decreases the likelihood of negative evaluations of the respondents’ life satisfaction while increasing the likelihood of positive evaluations. Specifically, for each additional child, the probability of being very dissatisfied with life decreases by 0.126 percentage points, the probability of being dissatisfied decreases by 0.393 percentage points, the probability of being neutral decreases by 1.544 percentage points, the probability of being satisfied increases by 0.239 percentage points, and the probability of being very satisfied increases by 1.824 percentage points.

For respondents of different genders, the marginal effect of increasing the number of children with the life satisfaction of male respondents is greater than that of female respondents (see Table 4). Specifically, for each additional child, it can reduce the probability of male respondents being very dissatisfied, dissatisfied, and neutral by 0.344, 0.539, and 2.796 percentage points, respectively. It can also increase the probability of them being satisfied and very satisfied by 0.471 and 3.208 percentage points, respectively, which is higher than the influence coefficient of the entire sample. The number of children only has a significant association with female respondents at the 10% level, and the coefficient is lower than that of male respondents. For each additional child, it can reduce the probability of female respondents being very dissatisfied, dissatisfied, and neutral by 0.072, 0.304, and 1.089 percentage points, respectively. It can also increase the probability of them being satisfied and very satisfied by 0.158 and 1.306 percentage points.

**Table 4:** Marginal effects of number of children with life satisfaction of UOALA

		Very Dissatisfied	Dissatisfied	Neutral	Satisfied	Very Satisfied
Number of children	Full sample	-0.001263***	-0.0039256***	-0.0154436***	0.0023912**	0.0182411***
		0.0004444	0.0012077	0.0045675	0.0009379	0.0053808
	Male	-0.0034383**	-0.005392***	-0.0279641***	0.0047098*	0.0320846***
		0.0013991	0.0019031	0.0086889	0.002454	0.0097533
	Female	-0.0007175*	-0.0030431*	-0.010886*	0.0015843*	0.0130623*
		0.0003983	0.001528	0.0053385	0.0009417	0.0064058
Number of sons	Full sample	-0.0005101	-0.0015818	-0.0062203	0.0009496	0.0073626
		0.0004651	0.0013975	0.0055036	0.0008751	0.006509
	Male	-0.0022027	-0.0034538	-0.0178953	0.002996	0.0205558
		0.0015261	0.0022136	0.0113652	0.0022757	0.0129612
	Female	-0.0001264	-0.0005351	-0.0019142	0.0002745	0.0023012
		0.0004191	0.0017633	0.0063126	0.000909	0.0075886
Number of daughters	Full sample	-0.0012098**	-0.0037657***	-0.0148078***	0.0022689**	0.0175143***
		0.0004773	0.0013785	0.0051949	0.0009948	0.0061441
	Male	-0.0028092**	-0.0044094**	-0.0227066**	0.0037171	0.0262081**
		0.0014302	0.0021447	0.0100133	0.0023363	0.0114445
	Female	-0.000813	-0.0034532**	-0.0123604**	0.0017886	0.014838**
		0.0004441	0.0017377	0.0060202	0.0010633	0.0072337

From the perspective of children’s gender, the marginal effect of the number of sons on respondents’ life satisfaction with life did not pass the significance test. However, in terms of coefficients, an increase in the number of sons will decrease the probability of respondents being very dissatisfied, dissatisfied, or neutral toward life, while increasing the probability of being satisfied and very satisfied. The marginal effect of the number of daughters on respondents’ life satisfaction with life is significant, as an increase in the number of daughters will decrease the probability of negative evaluations of life satisfaction by respondents, and increase the probability of positive evaluations, with a greater association with male respondents than female respondents. Specifically, for every additional daughter, the probability of respondents being very dissatisfied, dissatisfied, and neutral toward life decreases by 0.121, 0.377, and 1.481 percentage points respectively, while the probability of being satisfied and very satisfied increases by 0.227 and 1.751 percentage points respectively. For male respondents, the probability of being very dissatisfied, dissatisfied, and neutral toward life decreases by 0.281, 0.441, and 2.271 percentage points respectively, while the probability of being very satisfied increases by 2.621 percentage points. For female respondents, the probability of being dissatisfied and neutral decreases by 0.345 and 1.236 percentage points respectively, while the probability of being very satisfied increases by 1.483 percentage points, with a higher degree of association with male respondents than female respondents.

### 3.3 Association of the gender structure of children with life satisfaction

In this section, the explanatory variable is further expanded to the gender structure of children, which is derived by grouping children according to different genders based on quantity (2). The regression results of different gender structures of children with the life satisfaction of respondents’ lives are shown in Table 5. The regression results show that in some cases, UOALA who have both sons and daughters exhibit higher levels of life satisfaction, indicating a certain preference for having “both sons and daughters” among the older adults. Specifically, the estimated results of the model are as follows. In the first column, representing the regression on the entire sample, when UOALA have two children regardless of the gender structure, their life satisfaction significantly increases. Among them, the combination of having one son and one daughter passes the significance test at the 1% level, with a coefficient of 0.792. Having two daughters or two sons pass the significance tests at the 5% and 1% levels, with coefficients of 0.633 and 0.843, respectively. When there are two children, the life satisfaction of UOALA with both a son and a daughter is much higher than that of those with two sons, but slightly lower than that of those with two daughters. When UOALA have three children, the highest life satisfaction is observed when all three are daughters, passing the significance test at the 1% level, with a coefficient of 1.050. The next highest life satisfaction is observed when they have two sons and one daughter, and one son and two daughters, passing the significance tests at the 1% and 5% levels, with coefficients of 0.934 and 0.735, respectively. The lowest life satisfaction is observed when all three are sons, with a coefficient of 0.631. When UOALA have four or more children, having a mix of sons and daughters results in higher life satisfaction. The combination of three sons and one daughter, two sons and two daughters, and one son and three daughters pass the significance tests at the 1% level, with coefficients of 0.910, 1.012, and 0.924, respectively. Having four daughters only passes the significance test at the 10% level, with a coefficient of 0.691. The combination of both sons and daughters consistently passes the significance test and exhibits larger association coefficients, indicating a strong preference for having both sons and daughters among UOALA with multiple children.

**Table 5:** Regression analysis of the gender structure of children of respondents with life satisfaction.

	Full sample	Male	Female
	(1)	(2)	(3)
M	0.523* (0.284)	0.546 (0.415)	0.532 (0.406)
F	0.477 (0.292)	0.506 (0.461)	0.490 (0.406)
2M	0.633** (0.289)	1.065** (0.464)	0.502 (0.403)
1M1F	0.792*** (0.265)	0.732* (0.378)	0.829** (0.384)
2F	0.843*** (0.297)	0.984** (0.418)	0.822* (0.429)
3M	0.631* (0.334)	0.185 (0.577)	0.724 (0.444)
2M1F	0.934*** (0.273)	1.125*** (0.412)	0.887** (0.389)
1M2F	0.735*** (0.281)	0.622 (0.420)	0.800** (0.396)
3F	1.050*** (0.351)	1.165** (0.592)	1.016** (0.467)
4M	0.111 (0.415)	-0.392 (1.118)	0.203 (0.498)
3M1F	0.910*** (0.321)	1.088** (0.534)	0.868** (0.435)
2M2F	1.012*** (0.302)	1.825*** (0.476)	0.774* (0.416)
1M3F	0.924*** (0.301)	1.458*** (0.447)	0.777* (0.419)
4F	0.691* (0.373)	0.823 (1.076)	0.668 (0.461)
Control	Control	Control	Control
City	Control	Control	Control
N	2285	677	1608
R2_p	0.081	0.129	0.069
Chi2	407.651	192.715	244.906

The association of the gender structure of children with the life satisfaction of respondents of different genders is generally consistent. Additionally, the life satisfaction of male respondents is more influenced by the gender structure of their children compared to female respondents. In the regression analysis of male samples, when male respondents have two children, the combination of two sons or two daughters is positively associated with life satisfaction at the 5% significance level, with coefficients of 1.065 and 0.984 respectively. The combination of one son and one daughter has the lowest significance level, with the smallest coefficient of 0.732. Among male respondents with three children, the combination of two sons and one daughter, and the combination of three daughters, are significantly associated with higher life satisfaction at the 1% and 5% significance level respectively, with coefficients of 1.125 and 1.165. The association of having three sons with life satisfaction is not significant. When male respondents have four or more children, the combination of sons and daughters has a positive association with their life satisfaction. The combination of two sons and two daughters, and the combination of one son and three daughters, are positively associated with life satisfaction at the 1% significance level, with coefficients of 1.825 and 1.458 respectively. The combination of three sons and one daughter is positively associated with life satisfaction at the 10% significance level, with a coefficient of 1.088. The association of having four sons or four daughters with life satisfaction is not significant. In the third column for female samples, when female UOALA have two children, having one son and one daughter is positively associated with life satisfaction at a significance level of 5%, with a coefficient of 0.829. Having two daughters is positively associated with life satisfaction at a significance level of 10%, with a coefficient of 0.822. The association with life satisfaction for having two sons is not significant. When female UOALA have three children, having two sons and one daughter, or having three daughters is positively associated with life satisfaction at a significance level of 5%, with coefficients of 0.887 (2M1F), 0.800 (1M2F), and 1.016 (3F) respectively. The association with life satisfaction for having three sons is not significant. When female UOALA have four or more children, having both sons and daughters is positively associated with life satisfaction of male UOALA. Having three sons and one daughter is positively associated with life satisfaction at a significance level of 5%, with a coefficient of 0.868. Having two sons and two daughters, or having one son and three daughters, is positively associated with life satisfaction at a significance level of 10%, with coefficients of 0.774 and 0.777 respectively. The association between life satisfaction and having four sons or four daughters is not significant. In conclusion, in the case of having multiple children, having both sons and daughters is significantly associated with higher life satisfaction. However, in the groups with two and three children, older adults who have daughters have higher life satisfaction.

### 3.4 Association of the gender order structure of children with life satisfaction

The gender of children by birth order is a combination that arranges the genders of children based on their birth order, on top of the gender structure. Further investigation into the association of the gender of children by birth order with life satisfaction is conducted, and the regression results are shown in Table 6.

**Table 6:** Regression analysis of the gender structure of children of respondents with life satisfaction.

	Ologit		
	Full sample	Male	Female
	(1)	(2)	(3)
M	0.529* (0.285)	0.567 (0.421)	0.544 (0.410)
F	0.482* (0.293)	0.525 (0.470)	0.501 (0.410)
MM	0.641** (0.291)	1.087** (0.471)	0.516 (0.406)
MF	0.651** (0.276)	0.660* (0.401)	0.681* (0.400)
FM	1.008*** (0.291)	0.928** (0.462)	1.050** (0.408)
FF	0.854*** (0.298)	1.019** (0.426)	0.838* (0.433)
MMM	0.634* (0.336)	0.193 (0.592)	0.736* (0.447)
MMF	1.199*** (0.319)	1.062** (0.539)	1.265*** (0.435)
MFM	0.737** (0.318)	0.521 (0.466)	0.841* (0.444)
FMM	0.853*** (0.303)	1.893*** (0.544)	0.561 (0.412)
MFF	0.734** (0.345)	0.750 (0.541)	0.759 (0.467)
FMF	0.495 (0.323)	0.560 (0.585)	0.496 (0.435)
FFM	0.959*** (0.329)	0.590 (0.548)	1.140** (0.444)
FFF	1.058*** (0.352)	1.185** (0.598)	1.031** (0.470)
MMMM	0.114 (0.418)	-0.396 (1.163)	0.208 (0.503)
MMMF	0.737* (0.390)	0.665 (0.675)	0.786 (0.511)
MMFM	1.001** (0.490)	1.731** (0.786)	0.773 (0.627)
MFMM	0.893 (0.580)	0.211 (1.118)	1.160* (0.703)
FMMM	1.152** (0.486)	2.208** (0.868)	0.951 (0.593)
MMFF	1.757*** (0.464)	3.236** (1.420)	1.500*** (0.554)
MFMF	0.577 (0.551)	0.762 (1.083)	0.567 (0.676)
MFFM	0.484 (0.382)	1.509** (0.608)	0.175 (0.492)
FMMF	1.687*** (0.475)	3.345*** (0.817)	1.032* (0.593)
FMFM	0.854 (0.580)	1.214* (0.629)	0.736 (0.754)
FFMM	0.642 (0.407)	1.012 (0.638)	0.554 (0.528)
MFFF	0.794* (0.439)	0.298 (0.677)	1.132* (0.606)
FMFF	0.535 (0.459)	2.714*** (0.719)	0.146 (0.538)
FFMF	1.182*** (0.392)	1.933*** (0.585)	0.918* (0.532)
FFFM	1.059*** (0.382)	1.446*** (0.495)	0.987* (0.505)
FFFF	0.706* (0.375)	0.874 (1.112)	0.686 (0.465)
N	2285	677	1608
R2_p	0.086	0.146	0.074
Chi2	417.586	216.776	256.851

Specifically, in the first column, which represents the regression for the entire sample, it is found that respondents who have two or more children have a higher life satisfaction with daughters as their first child compared to sons. When respondents have two children, having a daughter as the first child is more associated with higher life satisfaction. The coefficients for having a son and a daughter (FM) and having two daughters (FF) are higher than having two sons (MM) or a son and a daughter (MF). When respondents have three children, combinations of children (MMM, MMF, MFM, FMM, MFF, FFM, and FFF) are significantly associated with higher life satisfaction. Among these combinations, MMF, FFM, FFM, and FFF are associated with higher life satisfaction at the 1% level, with all their coefficients exceeding 0.8. When respondents have four or more children, the combinations MMMF, MMFM, FMMM, MMFF, FMMF, FFMF, FFFF, and FFFF are significantly associated with higher life satisfaction. From these findings, it can be concluded that respondents have a higher life satisfaction when their first child is a daughter. This suggests that Hypothesis 3 needs to be revised. In recent decades, significant social changes, such as educational opportunities and family size, have made the eldest son’s responsibility for supporting parents in their old age no longer the only option for families. Daughters now play a stronger role in parents’ life satisfaction in their later years.

Columns (2) and (3) represent regression analysis on gender-specific samples. The results show that male respondents with three or more children have higher life satisfaction when their first child is a daughter. The association of the gender of children by birth order with life satisfaction of female respondents is relatively small. Specifically, for male respondents with two children, both the combination of two daughters (MM) and the combination of two sons (FF) are significantly associated with life satisfaction. For male respondents with three children, combinations such as two daughters and one son (FMM), three daughters (FFF), and two sons and one daughter (MMF) have a significant positive association, with the coefficient of FMM being greater than FFF and MMF. For male respondents with four or more children, the association of having a daughter as the first child with life satisfaction is significantly greater. The combinations are ranked in descending order of coefficients: FMMF > MMFF > FMFF > FMMM > FFMF > MMFM > MFFM > FFFM > FMFM > FFMM. The association of birth order with life satisfaction of female UOALA is significantly less pronounced. When female UOALA have 2 children, all combinations except MM show a significant association with life satisfaction. When they have 3 children, MMM, MMF, MFM, FFM, and FFF show a significantly positive association with life satisfaction. The association coefficients for life satisfaction are ranked as MMF>FFM>FFF>MFM>MMM. When female UOALA have 4 or more children, associations with life satisfaction are significantly positive for MFMM, MMFF, FMMF, MFFF, FFMF, and FFFM. The combinations with the highest association coefficients for life satisfaction are ranked as MMFF>MFMM>MFFF>FMMF. Overall, female UOALA who give birth to a daughter as their first child have higher life satisfaction. For male UOALA, having a daughter as their first child in larger families is associated with higher life satisfaction. The gender of children by birth order has relatively small differences in association with female UOALA’ life satisfaction.

## 4 Discussion

This study has the following limitations. Firstly, the study is based on cross-sectional data analysis rather than longitudinal tracking data. The questionnaire section is based on participants’ recollections, which may lead to recall bias and social desirability bias. Therefore, the study can only explore the correlation between fertility outcomes and life satisfaction among UOALA, rather than causal relationships. Secondly, previous research has found a significant association between “grandchild care” and older adults’ life satisfaction (22, 23). If older adults live close to their children, grandchild care may be possible, which could influence the exploration of its association with life satisfaction. Due to limitations in questionnaire design, we were unable to obtain relevant data on “grandchild care” to further investigate this issue. However, in the future, we can conduct related surveys to enrich and extend our research. Finally, due to limitations in research conditions, we were unable to obtain relevant data through a large-scale census. This study was conducted at a specific time and location. As the study discusses multiple child situations, there may be differences in data quantity due to different combinations of gender of children by birth order. Therefore, there may exist limitations regarding over-stretched data given the small sample size for various sub-groups, limiting its generalizability and applicability beyond the research context.

## 5 Conclusion

Based on micro-survey data from 2013 to 2015 in five major cities in China, this study compares the current situation of life satisfaction among urban older adults living alone (UOALA) with different characteristics of children. It analyzes the association of the number of children, the marginal effects of the number of children, their gender structure, and the gender of children by birth order with the life satisfaction of older parents, as well as gender differences. The conclusion are as follows.

The overall level of life satisfaction in UOALA in China falls between “Neutral” and “satisfied”. The number of children, their gender structure and gender of children by birth order all have varying degrees of association with their life satisfaction. Specifically, firstly, an increase in the number of children has a significant positive association with the life satisfaction of UOALA, especially for male UOALA. This suggests that even in urban areas, the traditional view of “more children, more blessings” still holds, and this view is more pronounced among older men.

Secondly, in terms of gender structure, when there are multiple children, having both sons and daughters has a significant positive association with the life satisfaction of UOALA. When there are four or more children, UOALA who have both sons and daughters have higher life satisfaction than those who have children of only one gender. This also reflects the modern continuation of the traditional gender view of childbearing. It is worth noting that, at the same level of the number of children, the life satisfaction of older parents who have only sons is the lowest. One possible explanation for this is the gender difference in the upbringing of children in China. Raising sons requires more financial and labor costs in terms of marriage expenses and grandchild care, which may lead to a decrease in parents’ life satisfaction in their later years.

Finally, regarding birth order structure, it was found that UOALA with a first-born daughter have higher life satisfaction, suggesting that the first-born daughter can promote the life satisfaction of older parents in their later years. This is especially evident among older men. Compared to the first-born son, the first-born daughter has a stronger association with the life satisfaction of older parents, which may be attributed to the modern continuation of traditional family caregiving patterns and the advancement of women’s social and family status. Urban women have more resources and, while continuing their responsibilities for caring for their aging parents, are able to provide increasing intergenerational support, thereby helping their older parents living alone to achieve greater psychological life satisfaction in their later years.

This research focuses on the issue of life satisfaction among UOALA. It has been found that older adults with multiple children have higher life satisfaction. However, as the first generation of only children in China reaches old age, a new family structure is emerging with a "4+2+1" pattern. This pertains to households that usually consist of four grandparents, two parents, and one only child, resulting in a greater prevalence of empty nests and solitary living among older adults. (24). The uniqueness, scarcity, and irreplaceability of only children make their parents face greater risks in old age, particularly in terms of mental care (25, 26). Therefore, in addition to establishing the moral responsibility of only children to support their parents mentally, methods such as “community mental care for the older adults” and “self-care of mental health in old age” have also been proposed (27). Furthermore, this research also found that UOALA with daughters tend to have higher life satisfaction. With societal progress and development, the preference for male offspring is gradually diminishing. The implementation of China’s three-child policy and the improved opportunities and rights for females in education, career, and family have contributed to this change. Daughters are usually considered gentle, considerate, and filial, often being more attentive in taking care of their parents. Therefore, more and more families are willing to have daughters, and this is closely related to the issue of gender equality in the new era.

## Data availability statement

The original contributions presented in the study are included in the article/supplementary materials, further inquiries can be directed to the corresponding author.

## Ethics statement

This article is based on secondary data. Ethical approval was not required for the study involving humans in accordance with the local legislation and institutional requirements. Written informed consent to participate in this study was not required from the participants or the participants’ legal guardians/next of kin in accordance with the national legislation and the institutional requirements.

## Author contributions

RZ: Writing – original draft, Writing – review & editing. JC: Writing – original draft, Writing – review & editing. XY: Writing – review & editing.
